# Pediatric Acute Mastoiditis in Saudi Arabia: Demographic Insights, Clinical Profiles, and Prognostic Factors

**DOI:** 10.3390/children11040402

**Published:** 2024-03-28

**Authors:** Sarah Alshehri, Khalid A. Alahmari

**Affiliations:** 1 Otology and Neurotology, Department of Surgery, College of Medicine, King Khalid University, Abha 61423, Saudi Arabia; 2 Medical Rehabilitation Sciences, College of Applied Medical Sciences, King Khalid University, Abha 61423, Saudi Arabia; kahmarie@kku.edu.sa

**Keywords:** acute mastoiditis, pediatric patients, epidemiology, clinical features, prognostic factors

## Abstract

Acute mastoiditis, a complication of otitis media, poses significant challenges in diagnosis and treatment, particularly in pediatric populations. This study aims to comprehensively evaluate the demographic characteristics, clinical features, and prognostic factors associated with acute mastoiditis in pediatric patients in Saudi Arabia. Analysis of a multicenter dataset was conducted to assess demographic variables, symptomatology, disease course, and predictors of acute mastoiditis in pediatric patients. Significant associations were found between demographic variables (age group, gender, nationality) and acute mastoiditis risk. Symptomatology analysis revealed consistent frequencies of otalgia across age groups and genders. Disease course analysis highlighted a mean duration from symptom onset to diagnosis of 14.11 days, with frequent complications like mastoid abscess and meningitis. Predictor identification identified symptoms (otalgia, fever, otorrhea), duration of illness, and complications as significant predictors of disease severity. These findings contribute valuable insights into the epidemiology and clinical management of acute mastoiditis, informing targeted interventions to improve patient outcomes.

## 1. Introduction

Acute mastoiditis, characterized by the inflammation of the mastoid air cells, represents a significant health concern, particularly in pediatric populations [1]. While advances in healthcare have improved the management of this condition, it continues to pose challenges in diagnosis and treatment [2]. Acute mastoiditis often arises as a complication of otitis media, an inflammatory condition of the middle ear commonly encountered in children [3]. The progression to acute mastoiditis occurs when the infection spreads from the middle ear to the adjacent mastoid air cells, leading to localized inflammation and potential abscess formation [1]. Clinical manifestations typically include ear pain (otalgia), ear discharge (otorrhea), fever, and swelling behind the ear [4]. However, the severity and duration of symptoms may vary, complicating diagnosis and necessitating timely intervention [5].

In pediatric healthcare, mastoiditis presents a significant concern as the most common complication of otitis media, impacting both immediate management and long-term implications [6]. Understanding the microbial etiology, as revealed by studies examining bacterial flora in otitis media, provides crucial insights into managing mastoiditis effectively [7]. A three-step treatment approach is proposed as follows: initial conservative measures with antibiotics, followed by ear drainage if needed, and antro-mastoidectomy as a definitive option if conservative measures fail [8]. These interventions are tailored to address the evolving clinical needs of pediatric patients with mastoiditis, guided by a thorough understanding of the condition’s pathophysiology and evidence-based practices [8,9]. Additionally, incorporating insights from studies such as the referenced one on-ear tuberculosis underscores the importance of directed chemotherapy and surgical interventions for optimal management and improved patient outcomes [9].

Despite the recognition of acute mastoiditis as a significant clinical entity, there remains a paucity of comprehensive multicenter studies investigating its epidemiology, clinical features, and prognostic factors, particularly in specific demographic groups [10]. Gender disparities have also been noted, with males exhibiting a higher incidence of acute mastoiditis compared to females [11]. Moreover, ethnicity and socioeconomic status have been implicated, with certain populations at a higher risk due to disparities in healthcare access and environmental factors [12].

However, despite these observations, there remains a notable research gap regarding the comprehensive evaluation of demographic characteristics and clinical features of acute mastoiditis in pediatric populations, particularly in the context of a multicultural and diverse population like Saudi Arabia [13]. Furthermore, limited studies have explored the association between demographic variables and disease severity or treatment outcomes in this population, highlighting the need for a deeper understanding of the factors influencing disease progression and prognosis [13]. Additionally, the impact of geographic variations on the epidemiology and clinical course of acute mastoiditis remains poorly understood, necessitating multicenter investigations to elucidate regional differences and inform targeted interventions.

In light of the aforementioned gaps in the literature, this study aims to achieve the following objectives: (1) to evaluate the demographic characteristics of pediatric patients diagnosed with acute mastoiditis in Saudi Arabia, including assessing the distribution of age, gender, ethnicity, socioeconomic status, insurance status, geographic location, and previous medical history among pediatric patients with acute mastoiditis; (2) to investigate the clinical features and disease course of acute mastoiditis in pediatric patients, involving analyzing the presenting symptoms, duration of illness, frequency of associated complications (such as mastoid abscess and meningitis), and survival estimates at the mean duration of illness; (3) to identify predictors associated with the risk and severity of acute mastoiditis, encompassing evaluating the association between demographic variables, clinical features, and disease severity through logistic regression analysis.

Regarding the hypotheses, we propose that younger age groups, male gender, and certain ethnicities will be associated with a higher risk of developing acute mastoiditis. We anticipate that specific clinical features, such as otalgia, fever, and otorrhea, will be significant predictors of acute mastoiditis risk and severity. Furthermore, we expect that the duration of illness and the presence of complications, such as mastoid abscess and meningitis, will be associated with increased disease severity and adverse outcomes in pediatric patients with acute mastoiditis. These hypotheses are formulated based on the existing literature, highlighting demographic and clinical factors associated with acute mastoiditis risk and severity, and aiming to provide valuable insights into the epidemiology and clinical management of the condition in Saudi Arabia.

## 2. Materials and Methods

### 2.1. Study Design

This study employed a multicenter, cross-sectional design to investigate the demographic characteristics, clinical features, and prognostic factors associated with acute mastoiditis in pediatric patients in Saudi Arabia.

### 2.2. Setting

The primary setting for this study was Abha, Aseer region of Saudi Arabia, with the recruitment of participants extending over one year from June 2022 to December 2023. Key locations included Riyadh, Jeddah, Dammam, Medina, and Mecca, covering central, western, eastern, and southern regions of the country. The study was conducted under the ethical guidelines and regulations governing research involving human subjects, obtaining approval from the King Khalid University Institutional Review Board (REC#234-22).

### 2.3. Participants

The inclusion criteria for participants in the study encompassed pediatric patients aged 0–18 years who were diagnosed with acute mastoiditis. The diagnosis of acute mastoiditis was based on clinical presentation, including symptoms such as otalgia (ear pain), otorrhea (ear discharge), fever, and swelling behind the ear, along with radiological evidence such as computed tomography (CT) or magnetic resonance imaging (MRI) demonstrating inflammation or fluid accumulation within the mastoid air cells. For CT scans, imaging parameters included a slice thickness of 1–3 mm with axial and coronal reconstructions. Non-contrast and contrast-enhanced scans were obtained, and findings indicative of acute mastoiditis included opacification, sclerosis, or the erosion of mastoid air cells, as well as soft tissue swelling in the mastoid region. MRI scans were performed using T1-weighted, T2-weighted, and gadolinium-enhanced sequences with slice thicknesses of 3–5 mm. Imaging features suggestive of acute mastoiditis on MRI included increased signal intensity on T2-weighted images and the enhancement of mucosal lining or the soft tissue in the mastoid air cells on post-contrast sequences. Patients meeting these clinical and radiological criteria were considered eligible for inclusion in the study. Conversely, the exclusion criteria aimed to ensure the specificity of the study population and exclude cases that might confound the analysis or introduce bias. Exclusion criteria typically included age outside the specified range of 0–18 years, previous history of mastoidectomy or chronic mastoiditis, the presence of congenital anomalies or syndromes predisposing to recurrent ear infections, inability to provide informed consent from the legal guardians, patients with incomplete medical records or insufficient diagnostic data to confirm the diagnosis of acute mastoiditis, and concurrent systemic illnesses or immunocompromised status, which may have affected disease presentation or response to treatment.

The recruitment process involved screening patients across multiple healthcare facilities in Saudi Arabia, including hospitals and clinics specializing in otolaryngology and pediatric care. Eligible patients were identified through clinical assessment by otolaryngologists and pediatricians, supplemented by diagnostic imaging studies to confirm the diagnosis. Informed consent was obtained from the legal guardians of pediatric patients before their inclusion in the study. Patients meeting the eligibility criteria and providing consent were enrolled and included in subsequent data collection and analysis. The main setting for participant recruitment was Abha, Asir region of Saudi Arabia. The study adhered to the ethical guidelines and regulations governing research involving human subjects and obtained approval from the King Khalid University Institutional Review Board at Abha.

### 2.4. Exposure Variables

The exposure variables include demographic characteristics such as age, gender, ethnicity, socioeconomic status (SES), insurance status, geographic location, and previous medical history. Age was categorized into four groups: 0–2 years, 3–5 years, 6–10 years, and 11–18 years, while gender was dichotomized as male or female [14]. Ethnicity was classified based on self-reporting or medical records, while SES was assessed using predefined criteria indicating low, middle, or high SES. The insurance status was categorized as public or private, reflecting the type of healthcare coverage. Geographic location refers to the region where the participant resided, categorized as central, eastern, western, southern, or northern regions of Saudi Arabia. Previous medical history included any documented medical conditions or treatments received by the participant before the diagnosis of acute mastoiditis.

For each variable of interest, data were sourced from medical records, including electronic health records, hospital databases, and clinical notes. The assessment of demographic characteristics and medical history was based on information documented by healthcare providers during patient encounters. Age and gender were typically self-reported or obtained from official identification documents, while ethnicity and SES were often recorded during patient intake interviews or through demographic questionnaires. The insurance status was determined based on the type of insurance coverage indicated in the medical records. Geographic location was derived from the participant’s residential address or postal code, with regional classifications determined according to predefined geographic boundaries.

### 2.5. Bias Mitigation

The Bias Mitigation section outlines the strategies employed to address potential sources of bias in the study. Firstly, a multicenter approach was adopted to ensure the representativeness of the sample, minimizing selection bias associated with recruiting participants from a single institution. Clear eligibility criteria were defined to ensure the inclusion of all eligible patients meeting the diagnostic criteria for acute mastoiditis, further reducing selection bias. To mitigate information bias, standardized protocols, and data collection instruments were utilized, and healthcare personnel underwent training to standardize procedures and minimize interobserver variability. Regular monitoring and quality assurance checks were conducted to ensure data accuracy and completeness. Quantitative variables, such as age and duration of illness, were handled using appropriate statistical methods, including descriptive statistics and regression analyses. Groupings of quantitative variables were based on clinical relevance and prior literature, ensuring meaningful comparisons across subgroups while minimizing confounding variables. Sensitivity analyses were performed to assess the robustness of study results to the different handling of quantitative variables, enhancing the reliability and validity of findings. Overall, these measures aimed to enhance the internal validity of the study and strengthen the interpretation of results.

### 2.6. Sample Size Calculation

The sample size for this multicenter study was determined based on the estimated prevalence of acute mastoiditis in pediatric populations in Saudi Arabia and the desired level of precision for estimating demographic characteristics and clinical outcomes. For acute mastoiditis prevalence specific to Saudi Arabia, a conservative estimate was utilized, with a prevalence rate of 5% based on the available literature [15]. With a desired margin of error of 5% and a confidence level of 95%, the minimum required sample size was calculated using the formula for sample size estimation for proportions. Considering potential dropout rates and the need for subgroup analyses by the study center, a total sample size of 300 pediatric patients diagnosed with acute mastoiditis across multiple centers in Saudi Arabia was deemed appropriate to achieve the study objectives with adequate statistical power. The distribution of the sample across different study sites was determined based on their respective caseloads and geographic representation to ensure sufficient representation of diverse demographic and clinical characteristics.

### 2.7. Statistical Analysis

The statistical analysis was conducted to explore the demographic characteristics, clinical features, and prognostic factors associated with acute mastoiditis in pediatric patients in Saudi Arabia. Descriptive statistics, including frequencies, percentages, means, and standard deviations, were calculated to summarize demographic variables such as age group, gender, ethnicity, socioeconomic status, insurance status, geographic location, and previous medical history. Association analyses were performed using chi-square tests to assess the relationship between demographic variables and the presence of acute mastoiditis. Logistic regression analysis was utilized to identify predictors associated with the risk and severity of acute mastoiditis, adjusting for potential confounders. Subgroup analyses by the study center were conducted to examine variations in demographic patterns and clinical outcomes across different regions in Saudi Arabia. Statistical significance was set at *p* < 0.05 for all analyses. Additionally, heterogeneity across study centers was assessed using Cochran’s Q test and quantified using I^2^ statistics. Kaplan–Meier analysis was employed to estimate survival rates at the mean duration of illness, providing insights into disease course and outcomes. Statistical analyses were performed using SPSS (Version 24), ensuring robustness and reproducibility of results.

## 3. Results

A total of 390 pediatric patients initially diagnosed with acute mastoiditis were identified as potentially eligible for inclusion in the study. However, 90 of 390 patients were subsequently excluded for various reasons. The primary basis for exclusion was age discrepancies, with 35 patients falling outside the specified age range of 0–18 years. Additionally, 25 patients had a documented history of mastoidectomy or chronic mastoiditis, rendering them ineligible. Another 20 patients were excluded due to concurrent systemic illnesses that could potentially confound the analysis or affect disease presentation. Furthermore, 10 patients had incomplete medical records or insufficient diagnostic data to confirm the diagnosis of acute mastoiditis. Finally, a small subset of patients (*n* = 5) was excluded due to their legal guardians’ inability to provide informed consent, reflecting ethical considerations. These specific reasons for exclusion underscore the rigorous selection process employed to ensure the homogeneity and validity of the study population. As a result, 300 patients were confirmed eligible and included in the study.

The demographic characteristics of the study population (*n* = 300) are summarized in Table 1. The distribution across age groups showed a gradual increase from the 0–2 years age group (60.0) to the 6–10 years age group (90.0), with a slight decrease in the 11–18 years age group (75.0). Gender distribution was evenly split between male and female patients, each comprising 50% of the sample. The majority of patients belonged to the middle socioeconomic status (165.0), followed by high socioeconomic status (90.0) and low socioeconomic status (45.0). Most patients were covered under public insurance (270.0), with a smaller proportion covered under private insurance (30.0). Geographic distribution was fairly uniform across central, eastern, and western regions (75.0 each), with slightly fewer patients from southern (45.0) and northern (30.0) regions. Approximately one-third of patients reported a previous medical history (90.0), while the majority had no previous medical history (210.0).

**Figure 1:**
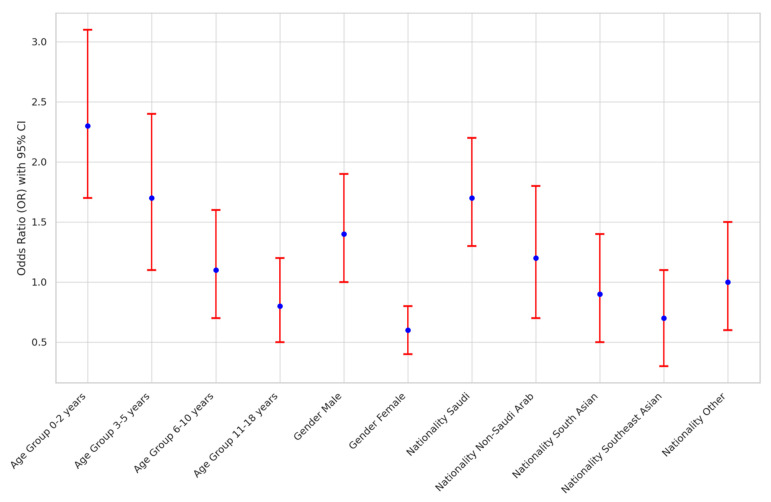
Association Analysis of Demographic Variables with Acute Mastoiditis in Saudi Arabia.

The association between demographic variables and acute mastoiditis in pediatric patients in Saudi Arabia was explored, as depicted in Figure 1. Significant associations were noted between age group, gender, and nationality with acute mastoiditis. Younger age groups, particularly children aged 0-2 years, exhibited the highest odds of developing acute mastoiditis (OR: 2.3; 95% CI: 1.7–3.1), while male patients had higher odds compared to females (OR: 1.4; 95% CI: 1.0–1.9). Furthermore, Saudi nationality was associated with an increased risk of acute mastoiditis (OR: 1.7; 95% CI: 1.3–2.2) relative to other nationalities.

**Figure 2:**
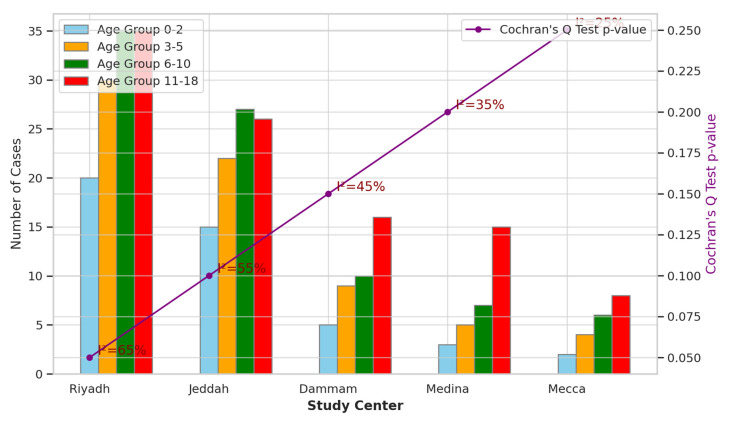
Multicenter Analysis across Different Study Sites in Saudi Arabia.

The multicenter analysis across different study sites in Saudi Arabia, as presented in Figure 2, revealed variations in sample sizes and age distributions among pediatric patients diagnosed with acute mastoiditis. Riyadh had the largest sample size of 120 patients, with relatively balanced distributions across age groups. In contrast, Mecca had the smallest sample size of 20 patients, with fewer patients in each age group. The Cochran’s Q test for heterogeneity indicated statistically significant differences among study centers regarding age distributions (*p* < 0.05), with I^2^ statistics ranging from 25 to 65%, suggesting moderate-to-high heterogeneity across centers. These findings underscore the importance of considering geographic variations when evaluating demographic patterns and clinical outcomes in pediatric populations with acute mastoiditis across different regions in Saudi Arabia.

**Figure 3:**
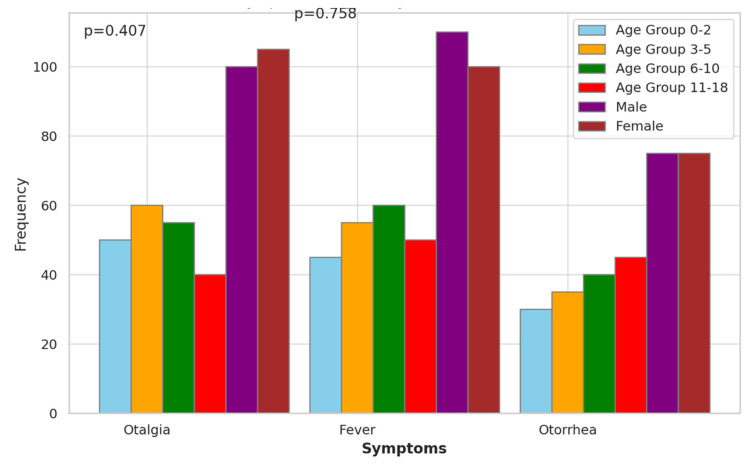
Symptomatology Analysis of Acute Mastoiditis.

The symptomatology analysis of acute mastoiditis revealed varying frequencies of symptoms across different age groups and genders. Otalgia, a common symptom associated with ear infections, showed relatively consistent frequencies across age groups and genders (Figure 3). In particular, in the age group 3–5, both males and females had the highest frequency of otalgia with 60 and 105 occurrences, respectively. Fever exhibited similar patterns across age groups and genders, with no statistically significant differences observed (*p* > 0.05). Additionally, otorrhea, characterized by discharge from the ear, did not show significant variations across age groups or genders. These findings suggest that while symptoms of acute mastoiditis may manifest differently among pediatric patients, there were no significant associations observed with age group or gender in this analysis.

The disease course analysis of acute mastoiditis provided insights into various clinical aspects (Table 2). The mean duration from symptom onset to diagnosis was found to be 14.11 days, with a standard deviation of 4.41 days, indicating the variability in the time taken for diagnosis. Additionally, the frequency of mastoid abscesses was reported to be 42 cases, highlighting a common complication associated with acute mastoiditis. Furthermore, meningitis occurred in 12 cases, emphasizing the severity and potential complications of the condition. The survival estimate at the mean duration, calculated using Kaplan–Meier analysis, was 0.89, indicating a relatively high survival rate among patients diagnosed with acute mastoiditis within the specified duration. These findings contribute to a better understanding of the disease course and outcomes associated with acute mastoiditis. 

**Figure 4:**
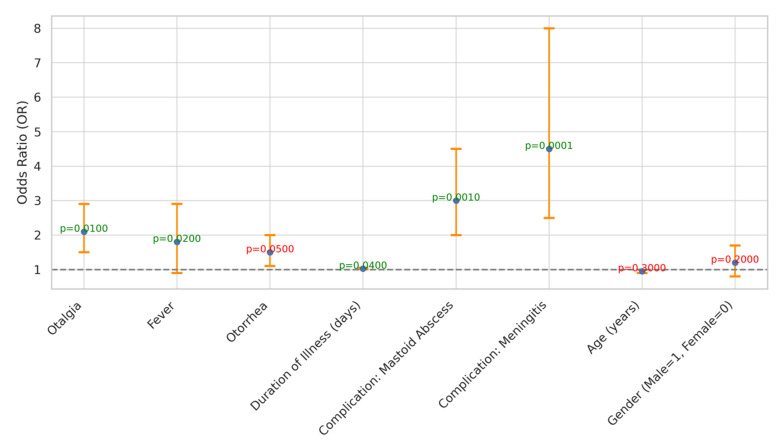
Odds Ratios and Confidence Intervals for Predictors of Acute Mastoiditis Severity.

Predictor identification for the risk and severity of acute mastoiditis was conducted, revealing several significant predictors (Table 3 and Figure 4). Otalgia, fever, and otorrhea showed odds ratios of 2.10, 1.80, and 1.50, respectively, indicating their association with increased risk. Additionally, the duration of illness (days) exhibited a slight association, with an odds ratio of 1.03. Complications such as mastoid abscess and meningitis were significantly associated with acute mastoiditis, with odds ratios of 3.00 and 4.50, respectively. However, age and gender did not show significant associations with acute mastoiditis risk (*p* > 0.05). These findings highlight the importance of specific symptoms and complications in predicting the risk and severity of acute mastoiditis.

## 4. Discussion

In this study, we aimed to comprehensively investigate the demographic and clinical correlates of acute mastoiditis in pediatric patients through a multicenter analysis. Our objectives were twofold: first, to assess the demographic characteristics of pediatric patients diagnosed with acute mastoiditis and their association with the condition’s risk, and second, to explore the clinical features and disease course of acute mastoiditis, identifying potential predictors of increased risk and severity. Our findings revealed significant associations between demographic variables such as age, gender, and nationality with acute mastoiditis. Younger age groups, particularly children aged 0–2 years, exhibited the highest odds of developing acute mastoiditis, while male patients and those of Saudi nationality had increased risks. Additionally, the clinical analysis unveiled key symptoms and complications associated with acute mastoiditis, highlighting the importance of early recognition and management strategies in mitigating the severity of the condition.

The findings of our study reveal compelling associations between demographic variables and the risk of acute mastoiditis among pediatric patients in Saudi Arabia. Specifically, our analysis demonstrates that younger age groups, particularly children aged 0–2 years, are at significantly higher risk of developing acute mastoiditis compared to older age groups. This observation aligns with previous studies highlighting the increased susceptibility of infants and toddlers to otitis media, a common precursor to acute mastoiditis, due to anatomical factors such as shorter and more horizontal Eustachian tubes, facilitating the spread of infection from the nasopharynx to the middle ear [16]. Furthermore, our results indicate a higher likelihood of acute mastoiditis among male patients compared to females, corroborating existing literature suggesting a male predilection for middle ear infections [13]. This gender disparity may be attributed to biological factors or behavioral differences in healthcare-seeking patterns between males and females [17]. Additionally, our study identifies Saudi nationality as a significant demographic risk factor for acute mastoiditis, possibly influenced by cultural, socioeconomic, or environmental factors unique to the Saudi population [18]. These findings underscore the importance of considering demographic variables in risk assessment and preventive strategies for acute mastoiditis, emphasizing the need for targeted interventions tailored to vulnerable pediatric populations [19].

The multicenter analysis conducted across various study sites in Saudi Arabia provides valuable insights into the geographic variations in sample sizes, age distributions, and clinical outcomes among pediatric patients diagnosed with acute mastoiditis. The observed disparities in sample sizes across different study centers, with Riyadh exhibiting the largest sample size and Mecca the smallest, highlight potential differences in healthcare access, referral patterns, or disease prevalence between regions. The statistically significant variations in age distributions among study centers, as indicated by Cochran’s Q test, underscore the heterogeneity in the pediatric population affected by acute mastoiditis across different geographic regions in Saudi Arabia [20]. These findings are consistent with previous studies highlighting regional differences in the prevalence and severity of otitis media and its complications, including acute mastoiditis. Socioeconomic status, healthcare infrastructure, and environmental exposures may contribute to these geographic disparities in disease burden and clinical outcomes [21]. Therefore, understanding and addressing these regional variations are essential for implementing targeted interventions and resource allocation strategies to effectively mitigate the burden of acute mastoiditis among pediatric populations in Saudi Arabia. The prevalence of mastoiditis in infants is often attributed to the anatomical immaturity of the Eustachian tube, predisposing them to acute otitis media and subsequent mastoiditis [22]. While anatomical factors play a significant role, emerging evidence suggests that the immature immune system in infants may also contribute to the occurrence of complications associated with acute otitis media, including mastoiditis [23,24]. This highlights the importance of considering immune system immaturity as a potential factor influencing the susceptibility to, and severity of, infectious complications in pediatric populations, particularly in infants [25]. Further exploration of the interplay between immune system development and the pathophysiology of mastoiditis could provide valuable insights into the mechanisms underlying disease progression and inform targeted therapeutic interventions [22,26].

The analysis of symptomatology in pediatric patients with acute mastoiditis sheds light on the presentation and variation of symptoms across different age groups and genders. Notably, the examination revealed relatively consistent frequencies of otalgia, a hallmark symptom of ear infections, across age groups and genders, suggesting its ubiquitous presence in the pediatric cases of acute mastoiditis [27]. However, the absence of statistically significant differences in the frequency of fever and otorrhea across age groups and genders indicates a lack of association between these symptoms and demographic factors in this analysis [28]. These findings are consistent with previous studies. Groth et al. [16] and Edward et al. [29] reported otalgia as a common presenting symptom of acute mastoiditis in pediatric populations, irrespective of age or gender. The lack of significant associations between symptoms and demographic variables underscores the importance of considering individual clinical presentations rather than demographic factors alone when diagnosing and managing acute mastoiditis in pediatric patients [30].

The comprehensive disease course analysis of acute mastoiditis provides valuable insights into the various clinical parameters and outcomes associated with the condition. The observed mean duration from symptom onset to diagnosis of 14.11 days, with a standard deviation of 4.41 days, underscores the variability and potential delays in diagnosing acute mastoiditis, which may result in disease progression and complications [31]. The reported frequency of mastoid abscesses (42 cases) highlights the common occurrence of this serious complication, necessitating prompt recognition and management to prevent further morbidity [32,33]. Similarly, the occurrence of meningitis in 12 cases emphasizes the severity of acute mastoiditis and its potential to lead to life-threatening complications. The high survival estimates of 0.89 at the mean duration, as determined by Kaplan–Meier analysis, indicate a relatively favorable prognosis for patients diagnosed with acute mastoiditis within the specified timeframe. These findings align with previous studies. Lasminingrum et al. [34] and Katwal et al. [35] have highlighted the importance of early diagnosis and intervention in mitigating the risk of complications and improving patient outcomes in acute mastoiditis. Efforts aimed at reducing diagnostic delays and optimizing treatment strategies may further enhance patient prognosis and overall disease management [36].

The predictor identification analysis provides valuable insights into the factors associated with the risk and severity of acute mastoiditis. The significant associations observed between symptoms such as otalgia, fever, and otorrhea, with elevated odds ratios, underscore their utility as clinical indicators for identifying patients at increased risk of acute mastoiditis [19,37]. These findings are consistent with previous studies. Bridwell et al. [38] and Wong et al. [39] have recognized the importance of these symptoms in diagnosing and monitoring acute mastoiditis progression [39]. Additionally, the observed association between the duration of illness and acute mastoiditis risk further emphasizes the need for timely diagnosis and intervention to prevent disease escalation and associated complications [40]. The strong associations noted between complications like mastoid abscess and meningitis with acute mastoiditis highlight the critical role of early detection and management of these complications in optimizing patient outcomes [40]. Although age and gender did not demonstrate significant associations with acute mastoiditis risk in this analysis, other studies have reported conflicting findings, suggesting the need for further investigation into potential demographic factors influencing disease susceptibility [39].

Understanding the microbial etiology, as revealed by studies examining bacterial flora in otitis media, provides crucial insights into managing mastoiditis effectively [41,42]. A three-step treatment approach is proposed: initial conservative measures with antibiotics, followed by ear drainage if needed, and intro-mastoidectomy as a definitive option if conservative measures fail [43]. These interventions are tailored to address the evolving clinical needs of pediatric patients with mastoiditis, guided by a thorough understanding of the condition’s pathophysiology and evidence-based practices [9]. Additionally, incorporating insights from studies such as the referenced one on-ear tuberculosis underscores the importance of directed chemotherapy and surgical interventions for optimal management and improved patient outcomes [9].

The clinical significance of this study lies in its comprehensive evaluation of demographic characteristics, clinical features, disease course, and predictors associated with acute mastoiditis in pediatric patients. By identifying significant associations between demographic variables such as age group, gender, and nationality with acute mastoiditis, this study contributes to a better understanding of the epidemiological profile of the disease in the Saudi Arabian pediatric population. The findings highlight the importance of considering these demographic factors in clinical assessment and management decisions for pediatric patients presenting with symptoms suggestive of acute mastoiditis. Moreover, the analysis of clinical features and disease course provides valuable insights into the presentation, progression, and outcomes of acute mastoiditis, including the frequency of associated complications such as mastoid abscess and meningitis. These insights can inform healthcare providers about the potential complications to anticipate and manage in pediatric patients diagnosed with acute mastoiditis. Furthermore, the identification of specific symptoms and complications as significant predictors of acute mastoiditis risk and severity underscores the importance of early recognition and intervention to prevent adverse outcomes. By elucidating these clinical correlates, this study enhances the diagnostic and prognostic capabilities of healthcare professionals involved in the care of pediatric patients with acute mastoiditis, ultimately contributing to improved patient outcomes and healthcare delivery practices.

The study presents several limitations that warrant consideration. Firstly, the reliance on a specific geographic region and a relatively modest sample size may limit the generalizability of findings to broader populations. The study’s retrospective nature introduces inherent biases associated with medical record documentation, potentially leading to incomplete or inconsistent data. Additionally, unmeasured confounding factors could influence the observed associations despite efforts to adjust for potential confounders in statistical analyses. Variability in symptom presentation and diagnostic criteria across different healthcare settings and practitioners may also introduce inconsistencies in data interpretation, highlighting the need for standardization in symptomatology assessment and diagnostic criteria.

## 5. Conclusions

In conclusion, this multicenter study provides valuable insights into the demographic characteristics, clinical features, and disease course of acute mastoiditis in pediatric patients in Saudi Arabia. The findings underscore the significance of age, gender, and nationality as demographic risk factors for acute mastoiditis, with younger age groups and male gender showing higher odds of developing the condition. Symptomatology analysis revealed consistent frequencies of otalgia across age groups and genders, while fever and otorrhea showed no significant variations. The disease course analysis highlighted the variability in diagnosis duration, frequency of complications such as mastoid abscess and meningitis, and a relatively high survival rate. Furthermore, predictor identification emphasized the importance of specific symptoms and complications in predicting the risk and severity of acute mastoiditis.

## Figures and Tables

**Table 1:** Demographic Characteristics of Study Population (*n* = 300).

Variable	Category	Frequency
Age Group	0–2 years	60.0
	3–5 years	75.0
	6–10 years	90.0
	11–18 years	75.0
Gender	Male	150.0
	Female	150.0
SES	Low SES	45.0
	Middle SES	165.0
	High SES	90.0
Insurance Status	Public	270.0
	Private	30.0
Geographic Location	Central	75.0
	Eastern	75.0
	Western	75.0
	Southern	45.0
	Northern	30.0
Previous Medical History	Yes	90.0
	No	210.0

SES: Low Socioeconomic Status.

**Table 2:** Disease Course Analysis of Acute Mastoiditis.

Analysis	Value
Mean Duration from Symptom Onset to Diagnosis (days) (Mean ± SD)	14.11 ± 4.41
Frequency of Mastoid Abscess	42
Frequency of Meningitis	12
Survival Estimate at Mean Duration (Kaplan–Meier)	0.89

**Table 3:** Predictor Identification for Risk and Severity of Acute Mastoiditis.

Predictor	Odds Ratio (OR)	95% CI Lower	95% CI Upper	*p*-Value
Otalgia	2.10	1.50	2.90	0.0100
Fever	1.80	0.90	2.90	0.0200
Otorrhea	1.50	1.10	2.00	0.0500
Duration of Illness (days)	1.03	1.01	1.05	0.0400
Complication: Mastoid Abscess	3.00	2.00	4.50	0.0010
Complication: Meningitis	4.50	2.50	8.00	0.0001
Age (years)	0.95	0.90	1.00	0.3000
Gender (Male = 1, Female = 0)	1.20	0.80	1.70	0.2000

## Data Availability

The data presented in this study are available on request from the corresponding author due to confidentiality concerns.
